# Keynes: die erneute Rückkehr des Meisters

**DOI:** 10.1007/s10273-020-2760-x

**Published:** 2020-10-24

**Authors:** Robert Skidelsky

**Affiliations:** Millbank House, 1 Millbank, SW1P 3JU London, UK

**Keywords:** B22, B31, E00

## Abstract

In der aktuellen Corona-Krise wiederholen sich die Muster früherer Krisen. Vor dem Hintergrund sinkender Produktion und steigender Arbeitslosigkeit versuchen Notenbanken und Staaten weltweit ihre Ökonomien vor einem größeren Absturz zu bewahren. Die Rezeptur für diese Stabilisierungspolitik basiert auf der Lehre des britischen Ökonomen John Maynard Keynes, die dieser vor dem Hintergrund der Großen Depression im Jahr 1936 in seiner „Allgemeinen Theorie der Beschäftigung, des Zinses und des Geldes“ beschrieb. Doch nicht nur in der Krise, auch darüber hinaus empfehlen sich die wirtschaftspolitischen Ansätze von Keynes für das 21. Jahrhundert.

## Abstract (en)

The current crisis repeats the patterns of previous crises. Against a backdrop of falling production and rising unemployment, central banks and governments around the world are trying to save their economies from a major crash. The formula for this stabilisation policy is based on the lessons of the British economist John Maynard Keynes, which he described after the Great Depression in 1936 in his “General Theory of Employment, Interest, and Money”. For the slump but also beyond, Keynes provided insightful policy recommendations that can be applied in the 21st century.

Mein Buch „Keynes: Die Rückkehr des Meisters" erschien im Herbst 2009, ein Jahr nach dem globalen Bankenkollaps von 2008 und den massiven Rettungsaktionen, die von Regierungen auf der ganzen Welt unternommen wurden. Damit gingen der Bailout eines bankrotten Bankensystems sowie umfangreiche geld- und fiskalpolitische Konjunkturprogramme einher. Anders als nach dem Wall-Street-Crash 1929 blieben die Regierungen in der Krise 2008/2009 nicht passiv. Ich gehe davon aus, dass durch diese Eingriffe eine weitere Große Depression verhindert werden konnte: Während die Wirtschaftsleistung nach dem Zusammenbruch 1929 über 13 Quartale in der Rezession blieb, beschränkte sich der Produktionsrückgang nach dem Zusammenbruch von 2008 auf vier Quartale.

## Mit dem Rücken zur Wand ist jeder ein Keynesianer

Bevor die Wirtschaft sich wirklich erholen konnte, wurden die Maßnahmen infolge der Krise 2008/2009 bereits wieder zurückgefahren. Die Angst vor einer möglichen Überschuldung ließ die Regierungen die öffentlichen Ausgaben kürzen und die „Rückkehr des Meisters" erwies sich als kurzlebig. „I guess everyone is a Keynesian in a foxhole", sagte Robert Lucas, der Hohepriester der Chicagoer Wirtschaft. Keynes war offenbar nur für Notfälle gedacht.

Heute ist das Muster ähnlich. Die Regierungen waren gezwungen, einen großen Teil ihrer Volkswirtschaften zu schließen, um die Ausbreitung der Coronavirus-Pandemie zu stoppen, und haben großzügig finanzielle Mittel bereitgestellt, um die Einkommen der Unternehmen und Millionen von arbeitenden Menschen zu sichern. Aber sie hofften, dass der Staatshaushalt bei der Wiedereröffnung der Wirtschaft entlastet wird, wenn der Aufschwung v-förmig verläuft. Damit die Staatsverschuldung nicht außer Kontrolle gerät, wird nun bereits wieder über Steuererhöhungen gesprochen.

Dies scheint vernünftig. Viele Unternehmer und Ökonomen halten das Marktsystem für grundsätzlich gesund. Es wird zwar von Zeit zu Zeit krank und braucht daher Medikamente, aber im Grunde ist es selbstheilend, wie der menschliche Körper. Deshalb sollte die Behandlung in Umfang und Dauer begrenzt sein. Dies gilt insbesondere angesichts der Risiken der politischen Medizin. Der britische Ökonom John Maynard Keynes (1883-1946) lehnte diese Analogie zwischen dem Marktsystem und dem selbstheilenden Körper ab und glaubte stattdessen, dass ein vom Staat unbeaufsichtigt gelassenes Marktsystem niemals gesund sein könne.

## Keynes für Anfänger

Die meisten, die vom Keynesianismus gehört haben, glauben, dass diese ökonomische Schule darauf abzielt, Haushaltsdefizite zuzulassen. Das ist falsch. Keynesianismus ist eine Wirtschaftstheorie, die ein ausgeglichenes Produktions- und Beschäftigungsniveau mithilfe des Staatshaushalts ermöglicht. Das kann entweder mit einem Haushaltsüberschuss, einem Haushaltsdefizit oder einem ausgeglichenen Haushalt einhergehen, je nachdem, wie die wirtschaftliche Lage sich gerade darstellt. Es ist nicht von sich aus erstrebenswert, den Staatshaus-

**Prof. Robert Skidelsky** ist Professor emeritus an der Universität Warwick und britischer Politiker.

halt auszugleichen. Vielmehr sollten konjunkturelle Auf- und Abschwünge ausgeglichen werden. Aber warum ist diese Balance so wichtig?

Keynes‘ revolutionäre Einsicht war, dass kapitalistische Marktwirtschaften nicht automatisch zur Vollbeschäftigung tendieren. Ihr Normalzustand tendiert zur Unterbeschäftigung, die sich bei einer ernsthaften Depression verschlimmert. Diese Behauptung schockierte die Ökonomen zur damaligen Zeit, deren Modelle lehrten, dass anhaltende Arbeitslosigkeit bei flexiblen Löhnen unmöglich sei. Keynes‘ Kollege aus Cambridge Arthur Pigou drückte diese Überzeugung so aus: „With perfectly free competition among workpeople and labour perfectly mobile ... there will always be at work a strong tendency for wage-rates to be so related to demand that everybody is employed“ (Pigou, 1933). Ausgehend von diesem Argument, müssen sich arbeitslose Arbeitnehmer dafür entscheiden nicht zu arbeiten, indem sie auf Löhne bestehen, die die Arbeitgeber ihnen nicht zahlen können. Als Keynes während der Großen Depression die Millionen von Arbeitslosen um sich herum sah, bemerkte er, dass etwas mit den Modellen nicht stimmen kann! Die Leute wollen nicht arbeitslos sein. Sie finden unabhängig vom Lohn keine Arbeit.

## Paradigmenwechsel

Die keynesianische Ökonomik beginnt mit einem Paradigmenwechsel. Keynes geht davon aus, dass Menschen arbeitslos sind, weil es keine Nachfrage nach Arbeit gibt. Doch diese Überlegung hatte die Wirtschaftswissenschaftler nicht wirklich überzeugt. Sie führten alle möglichen ausgefallenen Gründe an, um darzulegen, dass das, was wie unerwünschte Arbeitslosigkeit aussah, tatsächlich eine „Entscheidung für Freizeit“ war. Noch heute würde ich davon ausgehen, dass die meisten Ökonomen tief im Inneren glauben, dass fast alle Arbeitslosen einen Arbeitsplatz finden könnten, wenn sie es wirklich wollten oder wenn ihnen staatliche Sozialleistungen kein alternatives Einkommen bieten würden.

Aber warum erholen sich Volkswirtschaften nicht schnell von Zusammenbrüchen? Was ist mit der Idee der v-förmigen Erholung? Sicherlich kann ich, wenn ein Arbeitgeber mich nicht mit 500 Pfund pro Woche einstellen will, meine Lohnforderung so lange senken, bis es sich für ihn lohnt, mich einzustellen. Der orthodoxe Wirtschaftswissenschaftler hat eine fertige Antwort parat: Indem Arbeitnehmer auf unrealistische Löhne bestehen, nehmen sie sich selbst die Beschäftigung. Gegen diese Annahme führte Keynes zwei Gründe an, warum selbst flexible Löhne die Vollbeschäftigung nicht aufrechterhalten oder herstellen können.

## Kaufkrafttheorie der Löhne

Sein erstes Argument lautete, dass jeder Produzent auch ein Konsument sei: Mein Lohn ist dein Einkommen, denn mit meinem Lohn kaufe ich deine Waren. Wenn mein Lohn sinkt, sinkt dein Einkommen. Eine Senkung der Produktionskosten (sei es durch Lohnkürzungen oder durch Entlassungen von Arbeitnehmern) vertieft also einen Einbruch, in dem gleichzeitig die Kaufkraft und die Gesamtnachfrage verringert wird. Ein Einkommensrückgang in einem Teil der Wirtschaft reduziert die Produktion in einem anderen Teil usw. Es entsteht eine Abwärtsspirale, da sich die Arbeitslosigkeit in der gesamten Wirtschaft rasch ausbreitet. Schließlich wird die Kaufkraft erst auf einem deutlich niedrigeren Niveau stabilisiert, wenn die Menschen aufhören zu sparen. Aber nichts passiert, um den Konsum anzuregen und damit eine Erholung zu fördern. Nur ausgebildete Ökonomen kommen auf die verrückte Idee, dass der Weg in den Wirtschaftsaufschwung darin besteht, dass alle die Ausgaben kürzen.

## Vertrauen und Geldhaltung

Keynes‘ zweites Argument gegen die Idee der v-förmigen Erholung hatte mit der Geldhaltung zu tun. Es ist charakteristisch für eine Krise, dass Unternehmen ihr Geld horten bzw. ihre Barreserven aufstocken, statt zu investieren. Je größer diese „Liquiditätspräferenz“, desto höher ist der Zinssatz, den die Kreditgeber verlangen. Um die Produktion anzukurbeln, brauchen Kreditnehmer aber niedrigere, nicht höhere Zinsen. Wenn also das Vertrauen gering ist, bedeuten die höheren Zinssätze, die z. B. von den Banken verlangt werden, noch weniger Investitionen, weniger Konsum und weniger Beschäftigung.

So führen flexible Löhne und unflexible Zinssätze zu einer Vertiefung des Einbruchs. Anders als bei Robert Lucas bleibt die Wirtschaft ohne staatliche „Stimulierung“ in der Rezession. Aber die Mainstream-Ökonomik erlebt ein Comeback: Depressionen oder tiefe Rezessionen sind sehr seltene Ereignisse, wie die „schwarzen Schwäne“ von Nassim Taleb (2008). Es wäre absurd, das Wirtschaftsleben so zu organisieren, als stünde der nächste Einbruch unmittelbar bevor. Marktwirtschaften würden eine innere Stabilität aufweisen, sodass Krisen sehr seltene Ereignisse wären. Das hat Keynes jedoch bestritten: Schwarze Schwäne können jederzeit aus dem Nichts auftauchen.

Der Grund dafür ist laut Keynes, dass die Theorie der „sich selbst ausgleichenden“ Marktwirtschaft von der Vorstellung abhängt, dass jeder, und insbesondere Investoren die Zukunft genau vorhersagen können. Wenn sie den Wert der Vermögenswerte, die sie heute kaufen, in zehn Jahren genau berechnen könnten, würden sie niemals Dinge zu falschen Preisen kaufen. Wie Keynes (1937) schrieb: „The calculus of probability … was supposed to be capable of reducing uncertainty to the same calculable status as that of certainty itself." Aber das ist ein Mythos. „Actually, however, we have, as a rule, only the vaguest idea of any but the most direct consequences of our acts" (Keynes, 1936). Dies ist ein zweiter Paradigmenwechsel, der vielen Ökonomen die Augen öffnete. Dies hatte erhebliche Konsequenzen. Weil die Zukunft ungewiss ist, werden private Investitionen – die von der Erwartung künftiger Erträge abhängen – unstetig sein. Der Wohlstand hängt von den „animal spirits" der Menschen ab. Wenn sie sich zuversichtlich fühlen, stellen sie mehr Arbeiter ein; wenn sie pessimistisch sind, stellen sie weniger ein.

## Stabilisierungspolitik

Aus dieser Darstellung des Marktverhaltens ergeben sich zwei Schlussfolgerungen: Erstens, Zusammenbrüche sind immer möglich, weil die Zukunft ungewiss ist; und zweitens, wenn sie geschehen, gibt es keine „automatischen" Marktmechanismen, die einen schnellen Aufschwung gewährleisten. Deshalb muss der Staat als „Ausgleichsmechanismus" in der Marktwirtschaft wirken. Er steigert oder senkt die Nachfrage je nach Bedarf.

Diese zwei Dinge sollten Regierungen tun, und zwar nicht nur im Notfall, sondern dauerhaft:

1. Sie sollten die Investitionen stabilisieren. Dies können sie durch öffentliche Investitionsprogramme erreichen. Keynes (1936) schrieb: „I expect to see the state, which is in a position to [take] long views ... taking an ever greater responsibility for directly organising investment." Dies geschah in den 25 Jahren nach dem Zweiten Weltkrieg, doch seit den 1980er Jahren ist der Anteil des Staates an den Gesamtinvestitionen drastisch zurückgegangen, was die Instabilität der Investitionen erhöht.

2. Die Regierungen sollten eine „antizyklische" Politik verfolgen, um die Wirkung weiterer Schwankungen zu begrenzen. Das bedeutet, die Wirtschaft mit zusätzlichen Staatsausgaben anzukurbeln, wenn die privaten Ausgaben sinken, und sie zu drosseln, wenn diese steigen. Dies kann auf der Einnahmen- oder auf der Ausgabenseite oder auf beiden Seiten geschehen. Der „Multiplikator", der auf der „marginalen Konsumneigung" basiert, zeigt den Regierungen, welchen Gesamteffekt die zusätzliche oder reduzierte Nachfrage auf die Wirtschaft haben wird.

Diese beiden ausgleichenden Funktionen, öffentliche Investitionen und antizyklische Politik, sind notwendig, um Vollbeschäftigung und Stabilität in kapitalistischen Marktwirtschaften zu gewährleisten. Und je mehr Ressourcen kontinuierlich genutzt wurden, desto höher ist die Wachstumsrate und desto größer die soziale Zufriedenheit. Dies war – kurz zusammengefasst – die Botschaft von Keynes.

## Gegenargumente

Lassen Sie uns nun die wesentlichen Einwände gegen die keynesianische Theorie und Politik betrachten. Der erste ist, wie wir gesehen haben, dass Mainstream-Ökonomen glauben, dass Marktwirtschaften eine natürliche Stabilität aufweisen, anders als es Keynes annahm. Aber es gibt auch Argumente gegen einzelne keynesianische Instrumente.

1. Anti-keynesianische Ökonomen lehren, dass öffentliche Investitionen weniger effizient sind als private. Sie verdrängen (crowd out) sogar private Investitionen. Dies trifft zu, wenn alle Ressourcen der Wirtschaft voll ausgeschöpft werden. Wenn jedoch freie Kapazitäten vorhanden sind, können öffentliche Investitionen private Investitionen steigern (crowd in), indem sie die Gesamtnachfrage nach Gütern und Dienstleistungen erhöhen. Die meisten Regierungen haben die öffentlichen Investitionen nach den 1970er Jahren drastisch gekürzt. Das Wachstum wurde halbiert und die Arbeitslosigkeit stieg an. Tatsächlich wird ein Teil der öffentlichen Investitionen nicht effizient eingesetzt, aber das sollte mit der Ineffizienz von Arbeitslosigkeit abgewogen werden.

2. Monetaristische Ökonomen – Nachfahren von Milton Friedman – behaupten, dass die keynesianische „antizyklische" Politik zwangsläufig inflationär sein muss. Regierungen können den Konjunkturzyklus nicht steuern; und selbst wenn sie es könnten, würden sie im Bemühen Wählerstimmen zu gewinnen, so viel Geld drucken, dass es zu einer schleichenden und schließlich beschleunigten Inflation führen würde.

Diese monetaristische Kritik ist teilweise berechtigt. Aber auch hier müssen wir das keynesianische System mit der monetaristischen Alternative vergleichen. Monetaristen behaupten, dass die Märkte „antizyklisch stabil" seien, wenn es keine „Schocks" gäbe, da die Menschen rational in die Zukunft vorausschauen können. Der wichtigste „Schock", gegen den Vorkehrungen getroffen werden müssen, sind unerwartete Änderungen des Preisniveaus. Diese können die Menschen dazu verleiten, zu falschen Preisen zu handeln. Der Schlüssel zur wirtschaftlichen Stabilität ist daher eine niedrige und konstante Inflationsrate. Dies macht es erforderlich, dass die Kontrolle der Geldmenge oder der Zinssätze aus der Verantwortung der Politiker genommen und unabhängigen Zentralbanken übertragen wird. Dieses System wurde in den 2000er Jahren in den meisten Ländern getestet. Es hat 2008 bis 2009 einen wirtschaftlichen Zusammenbruch nicht verhindert.

Selbst die Politik der quantitativen Lockerung – die Überschwemmung der Wirtschaft mit Zentralbankgeld oder M0 in den Jahren 2009 bis 2012 – führte nach dem Zusammenbruch nicht zu einer v-förmigen Erholung. Der Trugschluss der Monetaristen besteht darin, dass die Geldmenge (zu der die erweiterte Geldmenge oder Bankkredite gehören) nicht direkt unter der Kontrolle der Zentralbank steht. Wie viel Bankkredit eine Ausweitung der Geldbasis bewirkt, hängt von Keynes' „animal spirits" ab. Ein sehr hoher Zinssatz kann einen Boom beenden, aber selbst ein negativer Realzinssatz führt möglicherweise nicht zu einer Erholung, wenn die „animal spirits" sich verdunkeln.

### Politikempfehlung

Die Antwort auf das Scheitern sowohl des altmodischen Keynesianismus als auch des neumodischen Monetarismus besteht nicht darin, die ausgleichende Rolle des Staates aufzugeben, sondern sie so automatisch wie möglich zu gestalten. Der Staat sollte sich zu zwei Dingen verpflichten: zu einem öffentlichen Investitionsprogramm und zu einer Arbeitsplatzgarantie im öffentlichen Sektor.

Das erste würde die Investitionsschwankungen enger begrenzen; die zweite würde einen Puffer an Arbeitsplätzen schaffen, der sich in einem Abschwung automatisch ausweiten und in einem Aufschwung reduzieren würde. Öffentliche Investitionen bedeuten nicht zwingend öffentliches Eigentum. Ein Teil davon könnte durch quasi-staatliche Institutionen wie öffentliche Investitionsbanken oder Unternehmen mit staatlicher Beteiligung getragen werden. Diese würden unter einem weit gefassten zentralstaatlichen Mandat stehen und damit nationale Ziele widerspiegeln, aber unternehmerische von politischen Entscheidungen trennen. Die öffentliche Arbeitsplatzgarantie würde zentral finanziert, aber mit Projekten, die lokal ausgewählt und verwaltet werden. Das Ergebnis beider Politiken gemeinsam wäre, dass zum ersten Mal seit der industriellen Revolution unerwünschte Arbeitslosigkeit abgeschafft würde.

Marxisten würden behaupten, dass ein solches aktualisiertes keynesianisches Programm nur ein Wunschtraum ist. Eine kapitalistische Wirtschaft braucht eine „Reservearmee der Arbeitslosen", um die Profite zu erhöhen und die Löhne zu senken. Nur eine vollständig sozialisierte Wirtschaft, so sagen sie, kann die Arbeitslosigkeit abschaffen und das Lohnwachstum aufrechterhalten. Tatsächlich wiesen die keynesianisch geführten kapitalistischen Volkswirtschaften von 1950 bis 1975 eine durchschnittliche Arbeitslosenquote von 2 % bis 3 % auf, halb so hoch wie seither, mit steigenden statt stagnierenden Löhnen, und die Inflation war nur geringfügig höher als während monetaristischer Führung.

Kein System der politischen Ökonomie ist vollkommen. Aber es sollte nicht im Vergleich mit einem idealen System beurteilt werden, sondern mit den realen Alternativen. Keynes machte sich daran, die Demokratie vor den beiden Herausforderern seiner Zeit – Faschismus und Kommunismus – zu retten. Er sagte, wenn wir angesichts der Massenarbeitslosigkeit mit der Laissez-faire-Politik fortfahren würden, würde die politische Freiheit nicht überleben. Wird das Problem aber richtig analysiert, könnte es möglich sein, die Krankheit zu heilen und gleichzeitig Effizienz und Freiheit zu bewahren.

Diese Überlegungen sind auch heute aktuell. Ich bezweifle, dass die westliche Bevölkerung über längere Zeit eine politische Ökonomie tolerieren wird, die dauerhafte Arbeitslosigkeit, häufige ökonomische Abstürze, stagnierende Löhne und extreme Ungleichheiten bei Vermögen und Einkommen mit sich bringt. Der Keynesianismus löst nicht alle wirtschaftlichen Probleme. Keynes (1936) schrieb, die beiden großen Fehler der kapitalistischen Gesellschaften seien das Versagen, Vollbeschäftigung zu schaffen, und seine arbiträre und ungleiche Verteilung von Einkommen und Vermögen. Er machte sich daran, den ersten Fehler zu überwinden, was das große Problem der damaligen Zeit war. Diese beiden Therapien mit dem Green New Deal zu verbinden, bleibt die größte wirtschaftliche Herausforderung unserer Zeit.
